# Detecting evolution of bioinformatics with a content and co-authorship analysis

**DOI:** 10.1186/2193-1801-2-186

**Published:** 2013-04-26

**Authors:** Min Song, Christopher C Yang, Xuning Tang

**Affiliations:** Department of Library and Information Science, Yonsei University, Seoul, South Korea; College of Information Science and Technology Drexel University Philadelphia, Philadelphia, PA 19104 USA

## Abstract

Bioinformatics is an interdisciplinary research field that applies advanced computational techniques to biological data. Bibliometrics analysis has recently been adopted to understand the knowledge structure of a research field by citation pattern. In this paper, we explore the knowledge structure of Bioinformatics from the perspective of a core open access Bioinformatics journal, BMC Bioinformatics with trend analysis, the content and co-authorship network similarity, and principal component analysis. Publications in four core journals including Bioinformatics – Oxford Journal and four conferences in Bioinformatics were harvested from DBLP. After converting publications into TF-IDF term vectors, we calculate the content similarity, and we also calculate the social network similarity based on the co-authorship network by utilizing the overlap measure between two co-authorship networks. Key terms is extracted and analyzed with PCA, visualization of the co-authorship network is conducted. The experimental results show that Bioinformatics is fast-growing, dynamic and diversified. The content analysis shows that there is an increasing overlap among Bioinformatics journals in terms of topics and more research groups participate in researching Bioinformatics according to the co-authorship network similarity.

**Electronic supplementary material:**

The online version of this article (doi:10.1186/2193-1801-2-186) contains supplementary material, which is available to authorized users.

## Background

Bioinformatics is an interdisciplinary field that involves research, development, or application of computational tools and methods for utilizing biological, medical, behavioral, or health data. Uzounis and Valencia (Ouzounis & Valencia^2003^) have provided a review of the early stages of the long history of the bioinformatics discipline. Recently, evolution and trends in bioinformatics research have been studied (Patra & Mishra^2006^; Bhaskar et al.^2006^; Perez-Iratxeta et al.^2007^). The field has been characterized as an emerging discipline that has arisen from the needs of biologists to utilize and help interpret the vast amounts of data that are constantly being gathered in genomic, proteomics and functional genomics research.

Studying a particular research field by its publication pattern is the realm of Bibliometrics analysis that is a research method used in library and information science. It utilizes quantitative analysis and statistics to describe patterns of publication within a given field or body of literature (Osareh^1996^). Bibliometric analysis has recently been applied to identify the development of the Bioinformatics field. Bansard et al. (2007) analyzed the bioinformatics and medical informatics literature to identify trends that are shared among both research fields to derive benefits from potential collaborative initiatives for their future. Their study shows that Bioinformatics and Medical Informatics are independent developments with limited overlaps although both undergo fast changes and apply advanced computer techniques to processing massive biological data. Huang et al. (2011) analyzed the citation patterns in bioinformatics journals by normalizing the journal impact factor available in Journal Citation Report (JCR) published by Thomson Reuters. Glänzel at al. (2009) retrieved the core literature in bioinformatics by combining textual components with bibliometric, citation-based techniques. Janssens et al. (2007) conducted a study to analyze the domain based on text mining and bibliometrics aided techniques, and aimed at improving classification of literature through the combination of linguistic and bibliometric tools. Ibáñez et al. (2009) developed a supervised learning technique to predict the possibility of a journal having a tool capable of predicting the citation count of an article within the first few years after publication would pave the way for new assessment systems. Jeong et al. (2009) investigated whether active members of conferences, who are conference organizers, keynote speakers, program committee members, etc, for scholarly events are representative of scholars' prominence by citation counts and H-index.

For the past decades, Bioinformatics has been expanded rapidly, and an understanding of the field of Bioinformatics becomes of paramount importance. The goal of this paper is to detect the trend and the knowledge structure of the field of Bioinformatics with the various approaches including tread analysis, the content and the co-authorship network similarity, Principal Component Analysis (PCA) of keywords and visualization of author Co-authorship. To our best knowledge, there are no studies analyzing Bioinformatics with co-authorship network analysis, a variation of social network analysis uses authors as the units of analysis and is constructed by connecting pairs of researchers authoring the same paper. This is different from author co-citation network constructed with two authors being co-cited by a paper. The co-citations of pairs of authors are considered as the variable that indicates their “distances” from each other. Co-authorship network has been widely used to study the structure of collaborations and the status of individual researchers (Liu et al.^2005^; Newman^2004^). For example, Cunninham (2001), Mutschke (2001), and Liu et al. (2005) have studied the structure of scientific collaborations of the digital library discipline. Most of the existing co-authorship network studies focused on topological features of static co-authorship network, including centrality, largest component, diameter, clustering coefficient, average separation, average number of collaborator etc. (Newman^2004^; Ding^2011^; Milojevi^2010^). The content similarity is helpful to understand if there are overlapping or related topics in different disciplines. The co-authorship network similarity helps us to understand the similarity of collaborative groups in different disciplines. A high co-authorship network similarity between two disciplines implies that there are collaborative groups participating in the scientific work on both disciplines. These two disciplines are likely to have common interests and/or highly relevant topics. However, the content similarity and co-authorship network similarity are not necessarily to be correlated. That is, it is probable that two disciplines have many common topics but the collaborative groups who work on these common topics in these two disciplines are not the same. The experimental results of our study verify this possibility. We analyze keywords extracted from the datasets by PCA to understand what topics constitute the core literatures. We also analyze the co-authorship network with visualization.

## Methods

### Problem definition and notation

We first present the definitions of publication and co-author network.

#### Definition 1 (Publication)

A publication can be a peer-reviewed paper from any conference or journal. It has attributes including title, year published, conference name (or journal name if it is published in a journal) and author list. A publication is the smallest unit in our study. We represent a publication *t*_*i*_ by a tuple$\;\left\{W_{t_{i}},N_{t_{i}},\mathrm{op}t_{t_{i}}\right\}$, where$W_{t_{i}}$ is the TF-IDF term vector of publication *t*_*i*_.$W_{t_{i}}=\left\{w_{1}^{t_{i}},w_{2}^{t_{i}},\ldots ,w_{\left|t_{i}\right|}^{t_{i}}\right\}\;$ is formed by the terms from *t*_*i*_ ‘s title, where$w_{j}^{t_{i}}$ denotes the TF-IDF score of the *j*^*th*^ term of *t*_*i*_. Similarly,$N_{t_{i}}$ is the co-author network (defined below) associated with the publication *t*_*i*_ and$\mathrm{op}t_{t_{i}}\;$ is the year published of publication *t*_*i*_.

#### Definition 2 (Co-author Network)

A co-author network associated with a publication *t*_*i*_ is a fully connected graph$N_{t_{i}}=<V_{t_{i}},E_{t_{i}}>$, where$V_{t_{i}}$ is a set of authors,$\left\{p_{1}^{t_{i}},p_{2}^{t_{i}},\ldots ,p_{\left|V_{t_{i}}\right|}^{t_{i}}\right\}$, co-authored in publication *t*_*i*_, and$E_{t_{i}}$ denotes the co-author relationships between authors in$V_{t_{i}}$. Every two co-authors are connected so that the co-author network is a fully connected network.

In this study, we are interested in the content similarity and social network similarity between BMC Bioinformatics and the other core Bioinformatics journals/conferences in a pre-specified period of time. We define *A*_*x*_ as a conference/journal *x* in the time period$t_{A_{x}}$. *A*_*x*_ can be represented by a triple$\;\left\{W_{A_{x}},N_{A_{x}},\mathrm{op}t_{A_{x}}\right\}$ where$W_{A_{x}}$ is the centroid of the TF-IDF term vectors of all publications of *x* in time period$t_{A_{x}}$,$W_{A_{x}}=\frac{1}{\left|A_{x}\right|}\underset{\mathrm{op}t_{t_{i}}\in \mathrm{op}t_{A_{x}}}{\sum }W_{t_{i}}$;$N_{A_{x}}$ is the aggregated co-authorship network of all$N_{t_{i}}$ of the publications from *x* with$\mathrm{op}t_{t_{i}}\in \mathrm{op}t_{A_{x}}$;$\mathrm{op}t_{A_{x}}$ is the pre-specified period of time. For example, let *A*_*x*_ represent the publications of BMC Bioinformatics in 2001. So that,$W_{A_{x}}$ is the centroid of the TF-IDF term vectors of all BMC Bioinformatics publications during 2001.$N_{A_{x}}$ is the co-authorship network integrating all co-authorship networks of the BMC Bioinformatics publications in 2001.$\mathrm{op}t_{A_{x}}$ then denotes the period of year 2001.

### Content similarity and social network similarity

As defined in the above section, the content of an individual publication is represented as a TF-IDF term vector and the content of a conference/journal is represented by the centroid of the term vectors of the publications from this conference/journal. Thus, the content similarity between two conferences/journals is the cosine similarity of their centroid term vectors, defined by$\cos\left(W_{A_{i}},W_{A_{j}}\right)$. Content similarity measures the topical commonality between two conferences/journals in terms of the keywords used in these domains. The scientific articles published in two totally different journals seldom use similar keywords; for example, the literature in criminology has very little keywords overlapping with the literature in cancer research. However, BMC Bioinformatics and Bioinformatics are two core journals in Bioinformatics with their own focus but there are also overlapping topics in these two journals. By using content similarity, we measure how similar the literatures of different journals are in terms of the vocabularies adopted by the corresponding authors.

As defined in Section 2, *A*_*x*_ is represented by the centroid of the term vectors of *A*_*x*_. Thus, the content similarity between *A*_*i*_ and *A*_*j*_  is measured by the cosine similarity of their centroid term vectors, defined as:

1 $$\cos\left(W_{A_{i}},W_{A_{j}}\right)=\frac{\sum_{k=1}^{N}w_{k}^{A_{i}}\times w_{k}^{A_{j}}}{\sqrt{\sum_{k=1}^{N}{\left(w_{k}^{A_{i}}\right)}^{2}}\times \sqrt{\sum_{k=1}^{N}{\left(w_{k}^{A_{j}}\right)}^{2}}}$$

where$w_{k}^{A_{i}}$ represents the k^th^ term of the centroid$W_{A_{i}}$. The numerator is the dot product of the TF-IDF term vectors of$W_{A_{i}}\;\mathrm{and}\;W_{A_{j}}$ and the denominator is the product of the Euclidean lengths of$W_{A_{i}}\;\mathrm{and}\;W_{A_{j}}$. The effect of the denominator is normalizing$W_{A_{i}}\;\mathrm{and}\;W_{A_{j}}$ to unit vector. The cosine similarity function measures the angle between two vectors in an n-dimensional space (n is the number of terms in the term vector). The smaller the angle is, the more similar the two TF-IDF term vector is.

Content similarity is capable of measuring the similarity of two journals in one aspect; however, it cannot measure the similarity in terms of the contributing authors and their collaboration networks. It is possible that the literatures of two journals have high content similarity but the contributing authors in these two journals can be quite different. For example, the topics covered in Bioinformatics and Medical Informatics are very similar; however, the contributing authors in these two domains can be very different. They are studying similar diseases but one is focusing on the prevention issue in the general public and another one is focusing on the findings in the treatment of these diseases. As a result, there is a high similarity in content but there may be a low similarity in terms of the contributing authors. In this work, we propose to measure the similarity of co-authorships networks to complement the content similarity analysis.

We propose the social network similarity analysis by considering the intersection of important authors involving in two journals*.* It is worth emphasizing that, rather than purely counting the number of overlapping authors in both journals, we assign a significant score to each individual author of a journal and measure the similarity by the significant scores of the contributing authors in two journals. Two co-authorship networks are considered as similar if the importance of the contributing authors is very close in both networks. If a contributing author is important in one co-authorship network but not as important in another co-authorship network, it will attribute to a lower social network similarity value. Two co-authorship networks will not be considered as similar only because they have a similar set of contributing authors.$\mathrm{Overlap}\left(N_{A_{i}},N_{A_{j}}\right)$ is defined as:

2 $$\mathrm{Overlap}\left(N_{A_{i}},N_{A_{j}}\right)=\frac{\sum_{P_{a}\varepsilon N_{A_{i}}\cap N_{A_{j}}}\min\left(\mathrm{SS}\left(P_{a}^{A_{i}}\right),\mathrm{SS}\left(P_{a}^{A_{j}}\right)\right)}{\sum_{P_{b}\varepsilon N_{A_{i}}\cup N_{A_{j}}}\max\left(\mathrm{SS}\left(P_{b}^{A_{i}}\right),\mathrm{SS}\left(P_{b}^{A_{j}}\right)\right)}$$

where$\mathrm{SS}\left(P_{k}^{A_{m}}\right)$ represents the significant score of the author *P*_*k*_ in *A*_*m*_. In this work, we employ degree centrality to measure the significance of an author. In a co-authorship network, edges are non-directional. There is no difference between in-degree and out-degree. Given a node *i*, its degree centrality (deg(*i*)) equals to the number of edges attached to this node which reflects the importance of this node in the given network. As a result, in this work$\mathrm{SS}\left(P_{k}^{A_{i}}\right)=\deg\left(P_{k}^{A_{i}}\right)$ in the co-authorship network$N_{A_{i}}$. The larger the degree centrality of an author in a co-authorship network is, the more co-authors this author have.$N_{A_{i}}\cap N_{A_{j}}$ denotes the intersection of authors in two co-authorship networks, and similarly$N_{D_{i}}\cup N_{D_{j}}$ denotes the union of authors in two co-authorship networks. From the definition above we can see that the larger the intersection of important authors, the higher the value of Overlap(•,•) is. For example, suppose$N_{A_{i}}$ represents the co-authorship network of journal *i* of year 2002 and$N_{A_{j}}$ represents the co-authorship network of journal *j* of year 2002,$\mathrm{Overlap}\left(N_{A_{i}},N_{A_{j}}\right)$ can be employed to quantify the co-authorship network similarity of these two journals in year 2002.

In general, the social network similarity over time may increase, decrease, or unchanged. When the social network similarity over time increases, it may due to the same group submitting to both journals in that particular year. It may also due to other reasons such as the authors with high degree centrality in one journal also have high degree centrality in another journal. In addition, the social network similarity is measured in each individual year. It does not accumulated over time, which means the similarity in 2010 does not necessary have more sufficient data than the previous year.

Based on the notations above, the similarity between *A*_*i*_ and *A*_*j*_ regularizing by *Social Network Analysis* is defined as

3 $$\begin{array}{l} \mathrm{score}\left(A_{i},A_{j}\right)=\epsilon \times \cos\left(W_{A_{i}},W_{A_{j}}\right)+\left(1-\epsilon \right)\\ \;\times \mathrm{Overlap}\left(N_{A_{i}},N_{A_{j}}\right) \end{array}$$

Where$W_{A_{i}}\;\mathrm{and}\;W_{A_{j}}$ are the centroids of TF-IDF vectors associated with *A*_*i*_*and A*_*j*_ respectively;$N_{A_{i}}\;\mathrm{and}\;N_{A_{j}}$ are social networks associated with *A*_*i*_*and A*_*j*_ respectively and 0 < *ϵ* < 1.

When *ϵ* is 1, it only measures the content similarity. When *ϵ* is 0, it only measures the social network similarity.

### PCA of keywords and co-author analysis in the field of Bioinformatics

First, we extract keywords from the datasets. Keywords of a journal within one year unit are selected based on their TF-IDF score. First of all, given a set of publications from a journal inside of a selected time interval, we extract the title for each publication and aggregated them together to represent the content of the journal of this time interval. Secondly, we conduct pre-processing on the content, including lowercasing, stemming, and removing stop words. Finally, we computed the TF-IDF score for each word of a journal within the time unit and ranked these words according to their TF-IDF score in descending order. Top words were returned as the keywords of the journal in this time unit. After we extract keyword lists, we apply PCA to the list. PCA in multivariate statistics is widely adopted as an effective unsupervised dimension reduction method and is extended in many different directions. The main justification of dimension reduction is that PCA uses singular value decomposition (SVD) which gives the best low rank approximation to original data in L2 norm.

For co-author analysis, we compute every pair of co-authors in the data collection. We then select top 500 co-author pairs to build an adjacency matrix. We used the betweenness centrality to calculate the node distance. Betweenness centrality is a measure based on the number of shortest paths between any two nodes that pass through a particular node. Nodes around the edge of the network tend to have a low betweenness centrality whereas a high betweenness centrality indicates that the individual is connecting various different parts of the network together.

## Results

In this section, we describe the dataset used in this study. As introduced in previous sections, the main focus of this paper is to analyze the trend and the content and social network similarity among BMC Bioinformatics and other core Bioinformatics literatures.

### Data collection

We constructed the dataset by extracting publication data from DBLP. The dataset consists of 16,061 peer-reviewed papers in Bioinformatics areas from 2001 to 2010. We extracted the *title*, *year of published*, *author list*, and *conference proceeding name* or *journal name* for each individual paper. It is important to note that our dataset covers the publications in four major conferences and four major journals of these two areas from 2001 to 2010. Formally speaking, we extracted papers from conferences including International Conference on Intelligent Systems for Molecular Biology (ISMB), Pacific Symposium on Biocomputing (PSB), International Conference on Bioinformatics & Computational Biology (BIOCOMP), and IEEE International Symposium on Biomedical Imaging (ISBI). In addition, we extracted papers from journals including Bioinformatics, Journal of Biomedical Informatics, PLoS Computational Biology and BMC Bioinformatics. We then empirically chose one year as a unit and then divided the dataset into 10 non-overlapping time intervals. It is worthy to mention that by the time that we collected our dataset some conference information was not completely available in DBLP. For example, DBLP only provides bibliography data for BIOCOMP from 2006 to 2010; ISMB 2009 and 2010 are unavailable; PLoS Computational Biology from 2001 to 2004 are unavailable; similarly, ISBI 2001 and 2003 are unavailable in DBLP. Last but not least, name disambiguation is an important yet very challenging pre-processing step in bibliometrics. DBLP employed a simple heuristic method to differentiate homonym persons. Besides automatic method, some daily manual efforts are also devoted by DBLP developers to further alleviate the ambiguous name problem. However, as mentioned in [DBLP-Some Lesson Learned], in many case, homonyms remain undetected. Name disambiguation is out of the scope of this paper. We simply used the disambiguated names provided by DBLP, although it may not be perfect. Table 1 and2 summarize the basic statistics of the data collection.

**Table 1:** Statistics of bioinformatics journals

	Bio	Bio	Bio	Bio	Bio	Bio	Bio	Bio	Bio	Bio
	2001	2002	2003	2004	2005	2006	2007	2008	2009	2010
# of Paper	204	280	443	561	767	599	660	600	727	692
# of Author	541	827	1413	1957	2665	2082	2409	2113	2731	2680
Author per Paper	2.65	2.95	3.18	3.48	3.47	3.47	3.65	3.52	3.75	3.87
	JBI	JBI	JBI	JBI	JBI	JBI	JBI	JBI	JBI	JBI
	2001	2002	2003	2004	2005	2006	2007	2008	2009	2010
# of Paper	36	38	46	45	48	32	74	96	101	119
# of Author	98	97	128	155	149	56	241	391	391	450
Author per Paper	2.72	2.55	2.78	3.44	3.10	1.75	3.25	4.07	3.87	3.78
	BMC	BMC	BMC	BMC	BMC	BMC	BMC	BMC	BMC	BMC
	2001	2002	2003	2004	2005	2006	2007	2008	2009	2010
# of Paper	9	40	66	209	414	633	613	745	729	737
# of Author	27	120	248	736	1465	2467	2416	2897	2863	2932
Author per Paper	3.00	3.00	3.75	3.52	3.53	3.89	3.94	3.88	3.92	3.97
	PLoS	PLoS	PLoS	PLoS	PLoS	PLoS	PLoS	PLoS	PLoS	PLoS
	2001	2002	2003	2004	2005	2006	2007	2008	2009	2010
# of Paper					71	168	252	287	375	414
# of Author					233	579	837	1005	1466	1683
Author per Paper					3.28	3.44	3.32	3.50	3.90	4.06

**Table 2:** **Statistics of bioinformatics conferences**

	ISMB	ISMB	ISMB	ISMB	ISMB	ISMB	ISMB	ISMB	ISMB	ISMB
	2001	2002	2003	2004	2005	2006	2007	2008	2009	2010
# of Paper	39	43	49	51	58	68	66	49		
# of Author	132	139	176	172	207	268	294	183		
Author per Paper	3.38	3.23	3.59	3.37	3.56	3.94	4.45	3.73		
	PSB	PSB	PSB	PSB	PSB	PSB	PSB	PSB	PSB	PSB
	2001	2002	2003	2004	2005	2006	2007	2008	2009	2010
# of Paper	59	63	61	55	51	55	47	62	51	51
# of Author	170	190	201	197	163	211	156	219	203	211
Author per Paper	2.88	3.01	3.29	3.58	3.19	3.83	3.31	3.53	3.98	4.13
	BIOC	BIOC	BIOC	BIOC	BIOC	BIOC	BIOC	BIOC	BIOC	BIOC
	2001	2002	2003	2004	2005	2006	2007	2008	2009	2010
# of Paper						87	119	163	146	146
# of Author						242	380	478	485	410
Author per Paper						2.78	3.19	2.93	3.32	2.80
	ISBI	ISBI	ISBI	ISBI	ISBI	ISBI	ISBI	ISBI	ISBI	ISBI
	2001	2002	2003	2004	2005	2006	2007	2008	2009	2010
# of Paper		267		391		350	346	411	361	366
# of Author		813		1206		1147	1159	1515	1253	1270
Author per Paper		3.04		3.08		3.27	3.34	3.68	3.47	3.46

### The trend in the co-authorship networks of Bioinformatics

**Figure 1:**
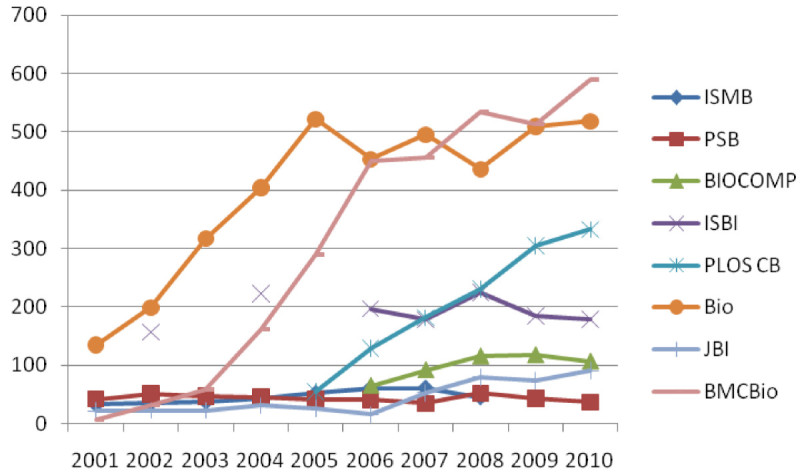
**The number of components in the co-authorship networks of Bioinformatics journals and conferences from 2001 to 2010.**

Before we examine the content similarity and social network similarity, we first look at the trend in the co-authorship networks of Bioinformatics journals and conferences. Figure 1 presents the number of components in the co-authorship networks of Bioinformatics journals and conferences over 10 years.

A social network component is a sub-graph that is connected within but disconnected from other sub-graphs. That means a node *n*_*i*_ in a social network component must have a path to all other nodes in the same social network component but do not have a path to any other nodes of other components.

A node *n*_*i*_ is part of a component *c*_*j*_ as long as there is a link between *n*_*i*_ and any one node of *c*_*j*_. Therefore, a component of a social network can be easily identified iteratively starting from a node and its links until no other nodes can be found.

As shown in Figure 1, the Bioinformatics journal has the largest number of component in 2001-2007 and has consistently increased during this time frame; BMC Bioinformatics and PLoS Computational Biology has also steadily increased in the number of components during 2001 and 2007. Journal of Medical Informatics and all four conferences (ISMB, PSB, BIOCOMP, and ISBI) have a small number of components and do not change as much as the other three journals. In 2007-2010, the number of components in BMC Bioinformatics is larger than those in Bioinformatics. It indicates that the number of collaborative groups in Bioinformatics journals except for JBI has increased substantially over a ten year period. BMC Bioinformatics shows the particularly rapid and consistent increase trend during the entire period of experiments. In BMC Bioinformatics, the biggest increase was made in 2003-2005. Note that the increase in the number of components was from 185 to 548. It is important to note that the number of components can be affected by the number of papers published in a period. When the number of papers published in a period is high, the probability of having a higher number of components is also higher.

**Figure 2:**
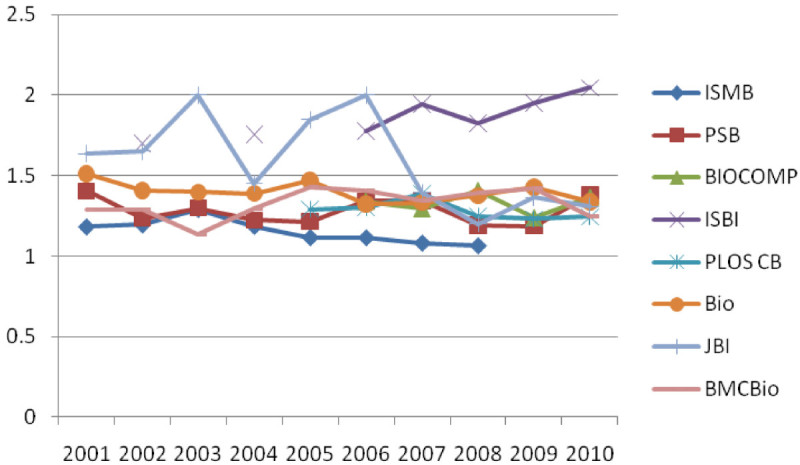
**The number of papers per component in the co-authorship networks of Bioinformatics journals and conferences from 2001 to 2010.**

Figure 2 and3 present the number of papers per component and the number of authors per component, respectively, in the co-authorship networks of four journals and four conferences in ten years. It is found that BMC Bioinformatics has the largest number of papers per component in the journal category and ISMB has the largest number of papers per component in the conference category. However, the differences observed in the number of papers in component were not statistically significant. In the number of authors per component, it is found that Bioinformatics has the largest number of papers per component followed by BMC Bioinformatics in the journal category. In the conference category, ISBI has the largest number of papers per component. The differences observed in the number of papers in component were not statistically significant.

**Figure 3:**
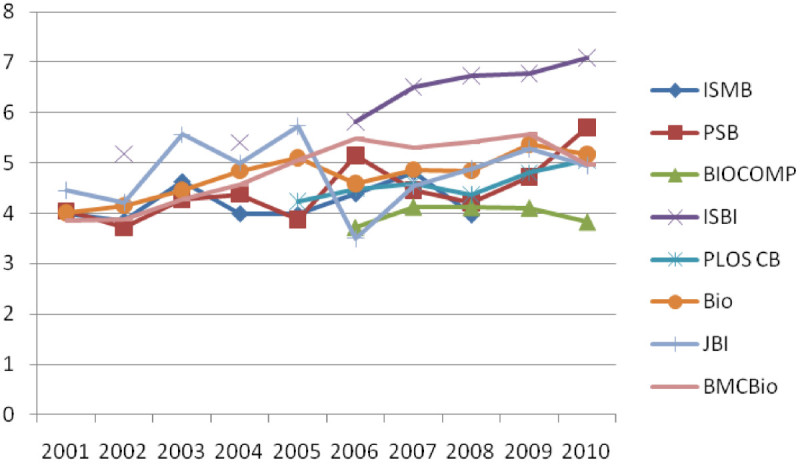
**The number of authors per component in the co-authorship networks of Bioinformatics journals from 2001 to 2010.**

In spite of the rapid growth in the number of components in BMC Bioinformatics, the number of papers per component and the number of authors per component in BMC Bioinformatics do not have the same increasing trend. Overall we observed that there exist similar patterns in the number of authors as well as in the number of papers in a component in both journals and conferences, which shows the moderate increase over 2001-2010. In addition, the experimental results show that there is a marginal deviation in the number of papers in component, which ranges from 2.93 to 3.63 on average. The number of authors in component shows a bigger deviation than in the number of papers whose range is from 4.21 to 7.06 on average. Both the lowest and the highest average number are of conference (BIOCOMP is lowest and ISBI is highest). The results of trend analysis indicate that a rapid growth of the number of components and an increase in the number of papers and authors within a component is not as high as an increase in the number of components. It implies that collaborative groups in Bioinformatics have been diversified and departmentalized which in turn presumably results from the fact that more researchers enter the field with specific research interests and background.

### Similarity analysis based on content and Co-authorship network

**Figure 4:**
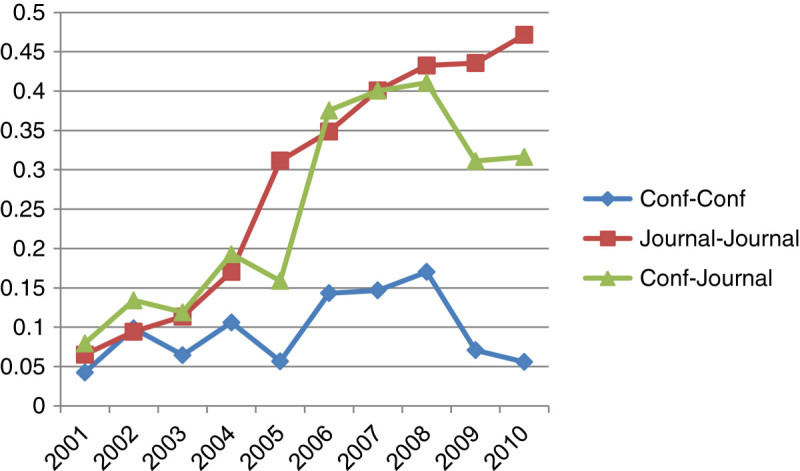
**Comparison of content similarity.**

We also conducted similarity analysis among conferences, conferences and journals, and journals in terms of content and co-authorship network similarity. Figures 4 and5 show relationships among these three categories by content similarity and co-authorship network similarity, respectively.

**Figure 5:**
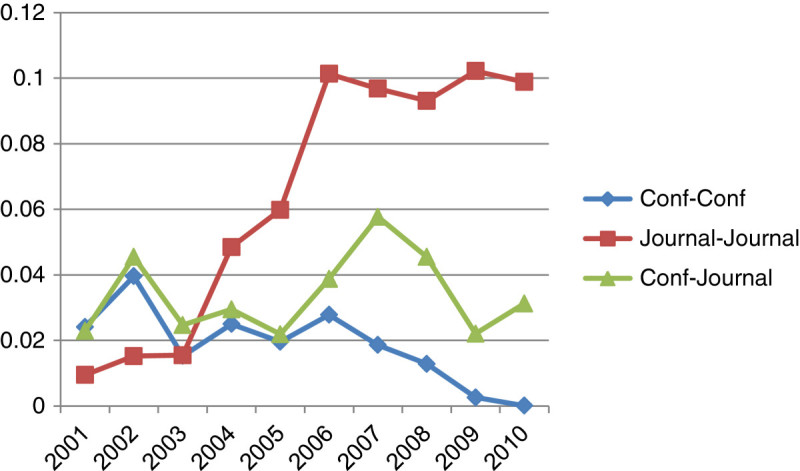
**Comparison of the co-authorship network.**

Figure 4 illustrates the content similarity among journals, conference and journals, and conferences in ten consecutive periods of time. It shows that there is a dramatic increase in content similarity among journals in periods of ten year. The similarity among journal and conferences has a sharp increase between 2005 and 2006, and then the curve shows fluctuation from 2006 to 2011. The relationship among conferences shows the low content similarity where the pick is below 0.2 in 2008.

**Figure 6:**
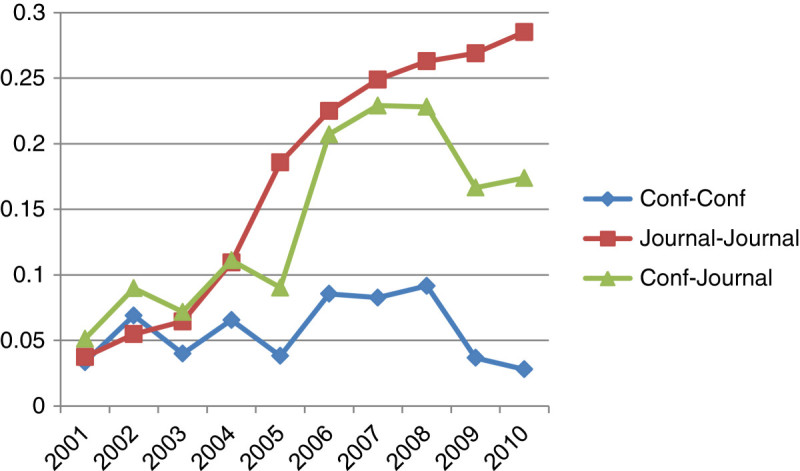
**Relationships among conferences and journals based on content and co-authorship network similarity.**

As illustrated in Figure 5, the overall co-authorship network similarity is considerably lower than the content similarity. Particularly, the co-authorship similarity among conferences is severely low. The similarity among journals is dramatically increased from 2003 to 2006 and then stabilized around at 0.1. The similarity between journals and conferences is fluctuated from 0.02 to 0.06 in ten year of the time span. Figure 6 presents the combination of content and co-authorship network similarity among journals, conference and journals, and conferences in ten consecutive periods of time. When both the content and the co-authorship network similarity are taken into consideration, the results show that the relationship among conferences shows the low similarity patterns as observed in both content and co-authorship network similarity. The experimental results indicate that 1) main themes and topics of conferences are somewhat different from topics covered in journals and 2) contribution groups of journals and conferences do not much overlap. This implies that the field of Bioinformatics becomes diversified. In addition, there is a high content similarity among journals whereas the co-authorship network similarity is not as high as the content similarity.

### Identification of keywords and key authors in the field of Bioinformatics

**Figure 7:**
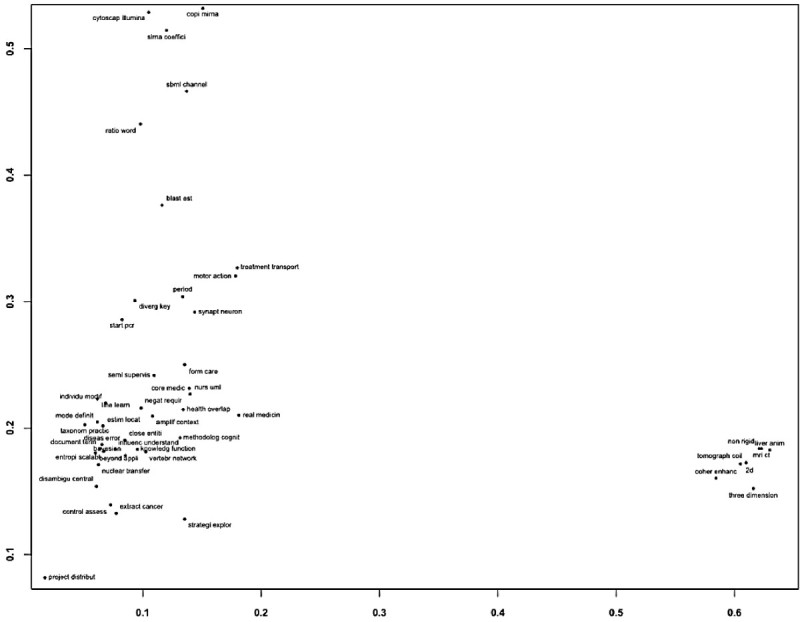
**The PCA results of keyword list.**

Figures 7 and8 show the results of applying PCA for keywords and visualization of the co-authorship network extracted from the datasets respectively. As illustrated in Figure 7, keywords are grouped into three discriminated clusters. In the upper left corner of two dimensional space, there are terms related to System Biology such as diverg (divergent), key (key), trace (trace), cytoscap (Cytoscape), illumina (Illumina), cgh (CGH), sbml (SBML), channel (Channel), interactom (Interactome), sirna (siRNA), coeffici (coefficient), and block (block). To reduce the term variations, we applied stemming for terms, and a term within parenthesis above is the original term appearing in the text. In the lower right corner, there appear terms related to medical imaging such as coher (coherent), enhanc (enhancement), tissu (tissue), mri (MRI), ct (CT), ultrasound (ultrasound), non-rigid (non-rigid), echocardiographi (echocardiography), morpholog (morphology), three-dimension (three-dimension) simultan (simultaneous). In the lower left corner, there is a dense cluster to which most terms are grouped. The core of the cluster consists of terms related to Bioinformatics including synapt (synaptic), neuron (neuron), plastic (plastic), algori (algorithm), care (care), protein-ligand (protein-ligand), database (database), mirna (miRNA), metagenom (metagenomics). In the right side of the cluster, there are terms related to medical informatics including vertebr (vertebrate), network (network), human (human), extract (extract), cancer (cancer), inform (information), semi-supervis (semi-supervised), gap (gap), diabet (diabetes), bayesian (Bayesian).

**Figure 8:**
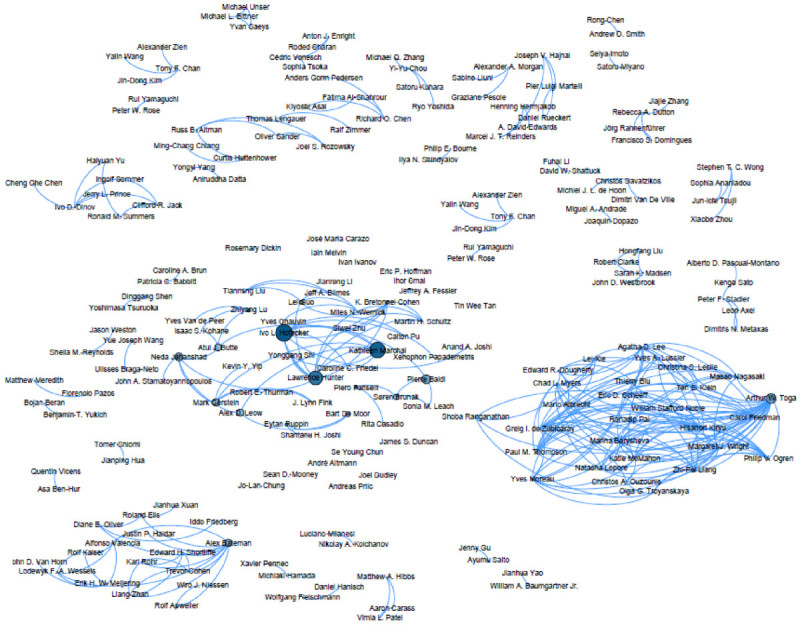
**Visualization of co-authorship analysis.**

Figure 8 shows visualization of co-authorship with the betweenness centrality measure. Several disconnected sub-graphs indicate that in Bioinformatics, a variety of segmented research group work on similar research topics in Bioinformatics. The biggest connected graph is located in lower right corner, which the main subject is pertinent to computational biology that includes researchers like Masao Nagasaki, Arthur W. Toga, and Lei Xie. This sub-graph also includes bio imaging researchers such as Paul M. Thompson and Agatha D. Lee. A sparsely connected sub-graph in the middle includes researchers like Ivo L. Hofacker who is interested in RNA Bioinformatics, Kathleen Marchal in Molecular Biology, and Lawrence Hunter in Computational Pharmacology. Broadly speaking, researchers located in the middle sub-graph have a specialty in computational biology.

The experimental results show that Bioinformatics is fast-growing, dynamic and diversified which confirms the findings by Huang et al. (2011) and Bansard et al. (2007). In addition, we have gained interesting findings from the experimental results. Our analysis shows that there is a substantial growth of collaborative groups in journals. It can be attributed to the solid increase of papers published in these journals. Such trend of growth is not observed in conferences. With respect to content similarity, the comparison among journals indicates a steady increase in content similarity in ten consecutive periods of time. This implies that the works published in these journals become more similar than before. From the perspective of co-authorship network similarity, no uniform pattern in three comparisons was observed. It is found that the social network similarity between conferences is very low and the similarity between conferences and journals is also low. The co-authorship network similarity among journals shows a steady increase until 2006 and then saturated. The content similarity between journals is relatively high and yet the co-authorship network similarity between these journals is moderately low. That means the collaborative groups are contributing very similar work to Bioinformatics journals but the contributing groups are not completely the same in these journals. It can be attributed to the different properties of the communities in these journals. In the future, it will be interesting to identify the properties of these two communities in order to understand how these two journals are different.

## Conclusions

The scientific literature is continuously developing. To gain a better understanding of such development, we conduct the trend analysis, the content and the co-authorship analysis, and PCA of keywords and visualization of co-authorship in the Bioinformatics research domain. The content similarity helps us to understand the development of topical similarity between different journals/conferences and the co-authorship network similarity helps us to understand the similarity of collaborative groups between different journals/conferences. In this work, we find that the field of Bioinformatics keeps growing. More researchers enter the field and collaborate with others although the collaboration rate is not as high as the publication growth in the field. It is also found that bioinformatics related journals are highly similar in terms of contents. It is interesting to note that there is the moderate increase in content similarity between conferences and journals but have fluctuation in terms of co-authorship network similarity. It indicates that both journals and conferences cover some overlapping topics but the contributing collaborative groups are dynamic and quite not similar. In addition, we find that there are three distinct clusters by PCA that is applied to the keyword list. Visualization of the co-authorship network reveals that several disjoint research groups that study the similar topics. This implies that the community is big and sparse so that they do not have a chance to collaborate with each other. Another interpretation is that it is the closed community so that the collaboration among different research groups does not frequently occur.

This work illustrates how content similarity and co-authorship network similarity supplement each other in bibliometric studies, which is useful in understanding the development of scientific literature of Bioinformatics. In the future, we plan to further investigate the co-authorship network similarity across consecutive periods of time so that we may understand the development of collaborative groups within a discipline. For example, we may understand if there is any dominating collaborative group and how such group is developing in consecutive periods of time.

## Authors’ original submitted files for images

Below are the links to the authors’ original submitted files for images. Authors’ original file for figure 1 Authors’ original file for figure 2 Authors’ original file for figure 3 Authors’ original file for figure 4 Authors’ original file for figure 5 Authors’ original file for figure 6 Authors’ original file for figure 7 Authors’ original file for figure 8
