# Supplementary figures and images for: Detecting evolution of bioinformatics with a content and co-authorship analysis
Source: Springerplus. 2013 Apr 26;2(1):186. doi: 10.1186/2193-1801-2-186 (PMC3661075; doi:10.1186/2193-1801-2-186)

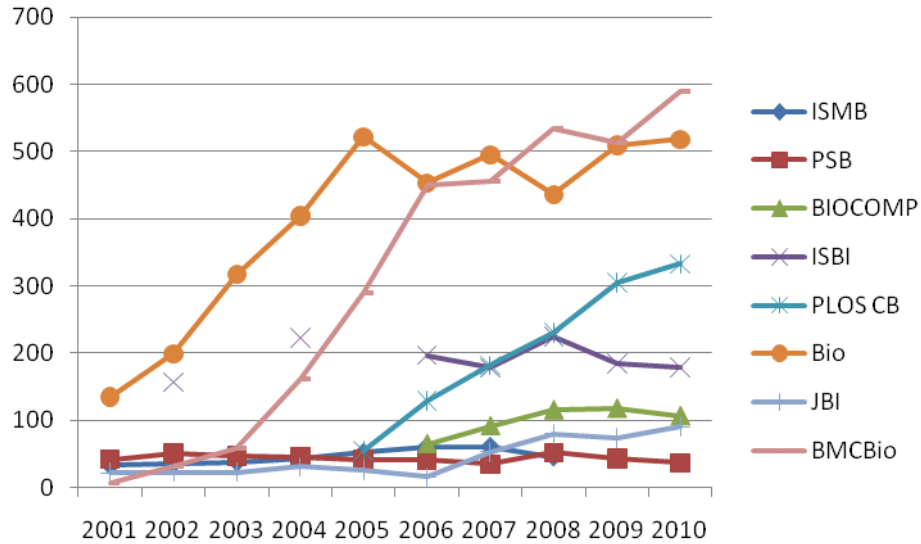

Supplement: Supplementary file 1 — Authors’ original file for figure 1 [file 40064_2012_267_MOESM1_ESM.pdf]

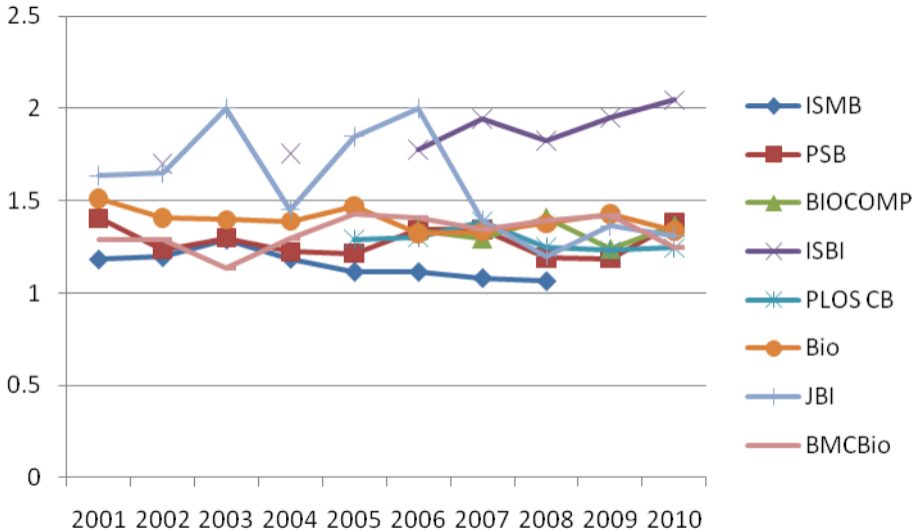

Supplement: Supplementary file 2 — Authors’ original file for figure 2 [file 40064_2012_267_MOESM2_ESM.pdf]

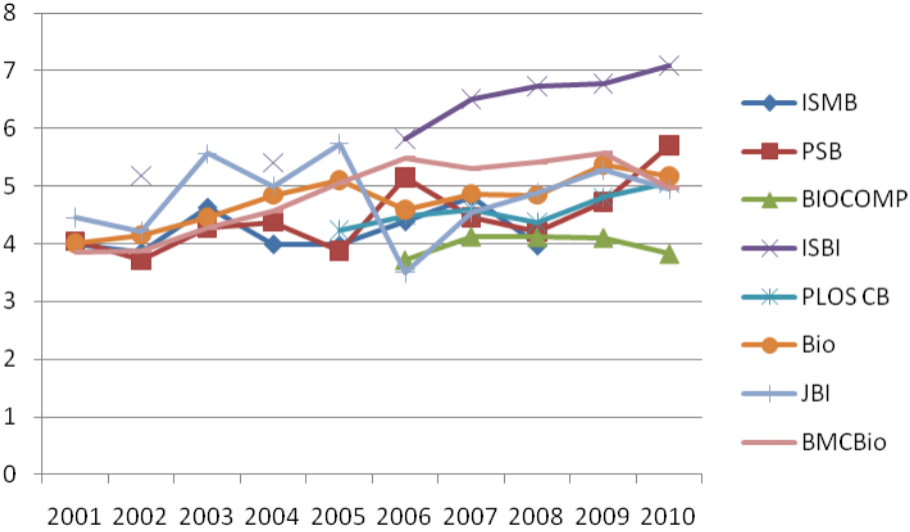

Supplement: Supplementary file 3 — Authors’ original file for figure 3 [file 40064_2012_267_MOESM3_ESM.pdf]

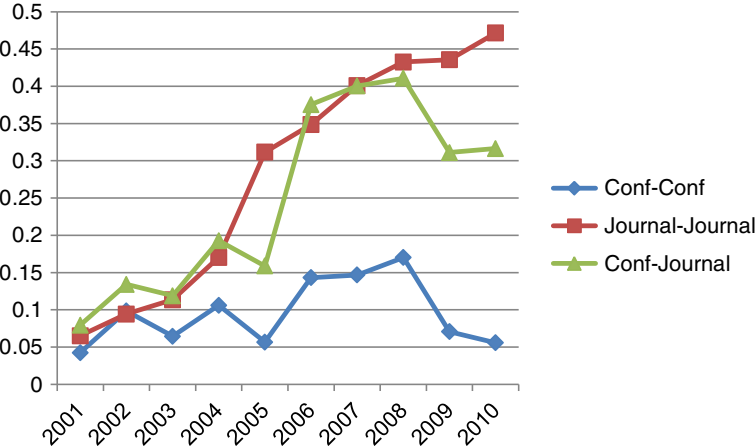

Supplement: Supplementary file 4 — Authors’ original file for figure 4 [file 40064_2012_267_MOESM4_ESM.pdf]

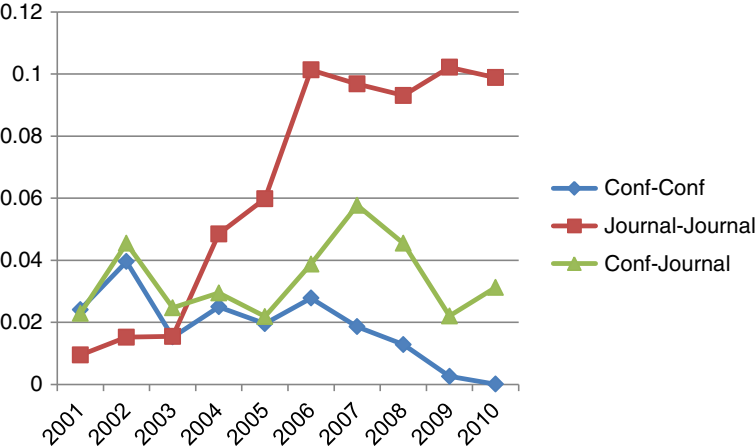

Supplement: Supplementary file 5 — Authors’ original file for figure 5 [file 40064_2012_267_MOESM5_ESM.pdf]

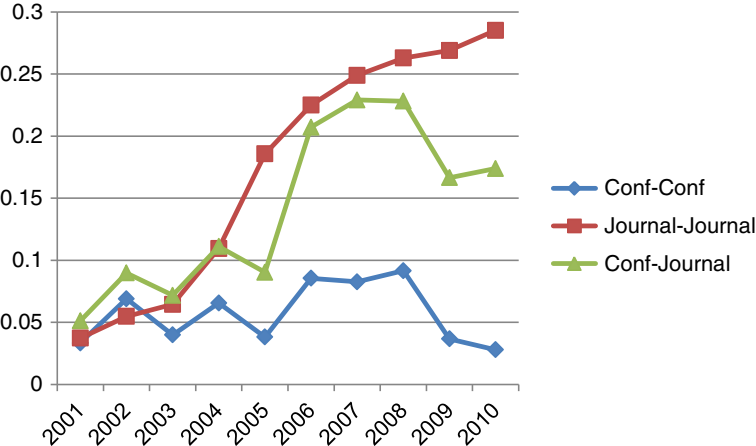

Supplement: Supplementary file 6 — Authors’ original file for figure 6 [file 40064_2012_267_MOESM6_ESM.pdf]

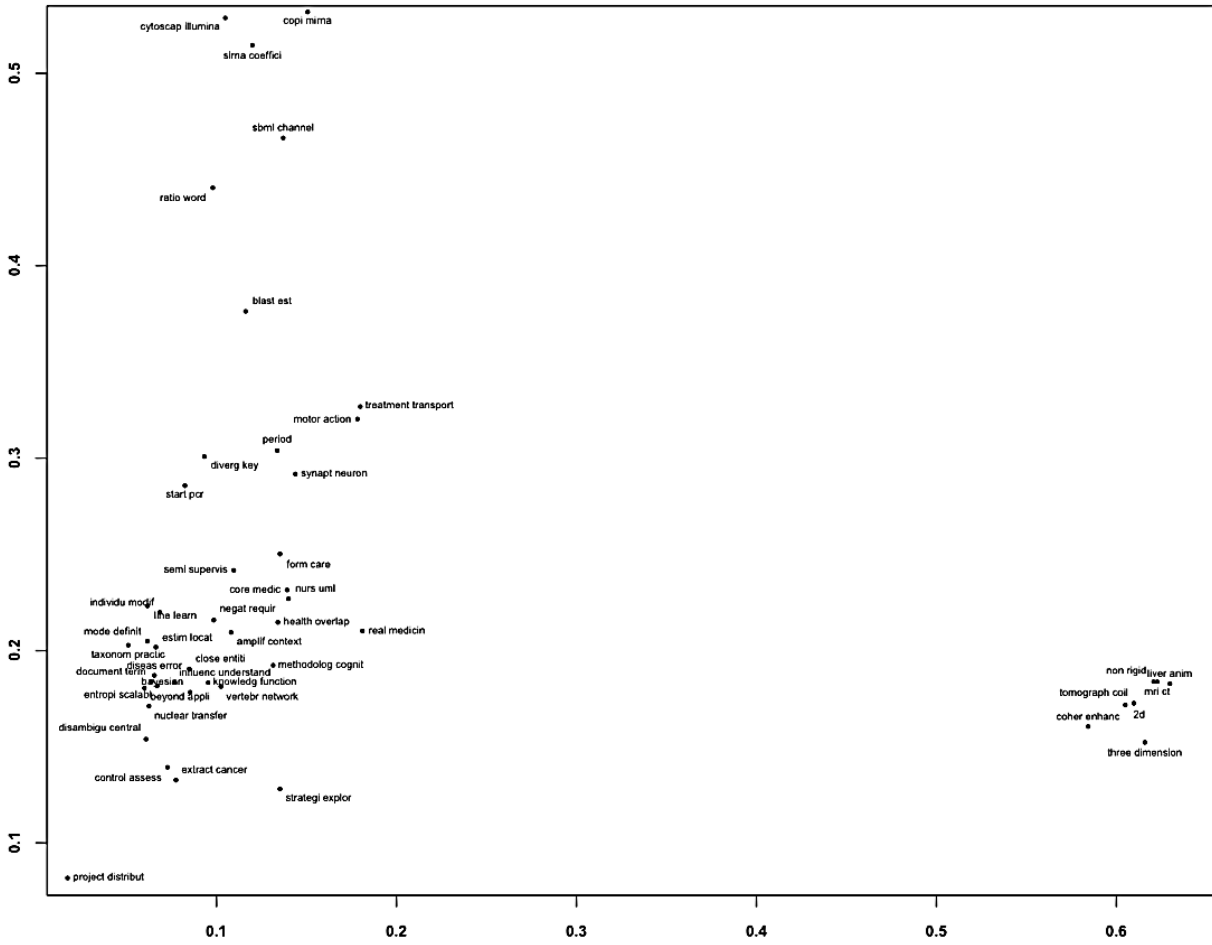

Supplement: Supplementary file 7 — Authors’ original file for figure 7 [file 40064_2012_267_MOESM7_ESM.pdf]

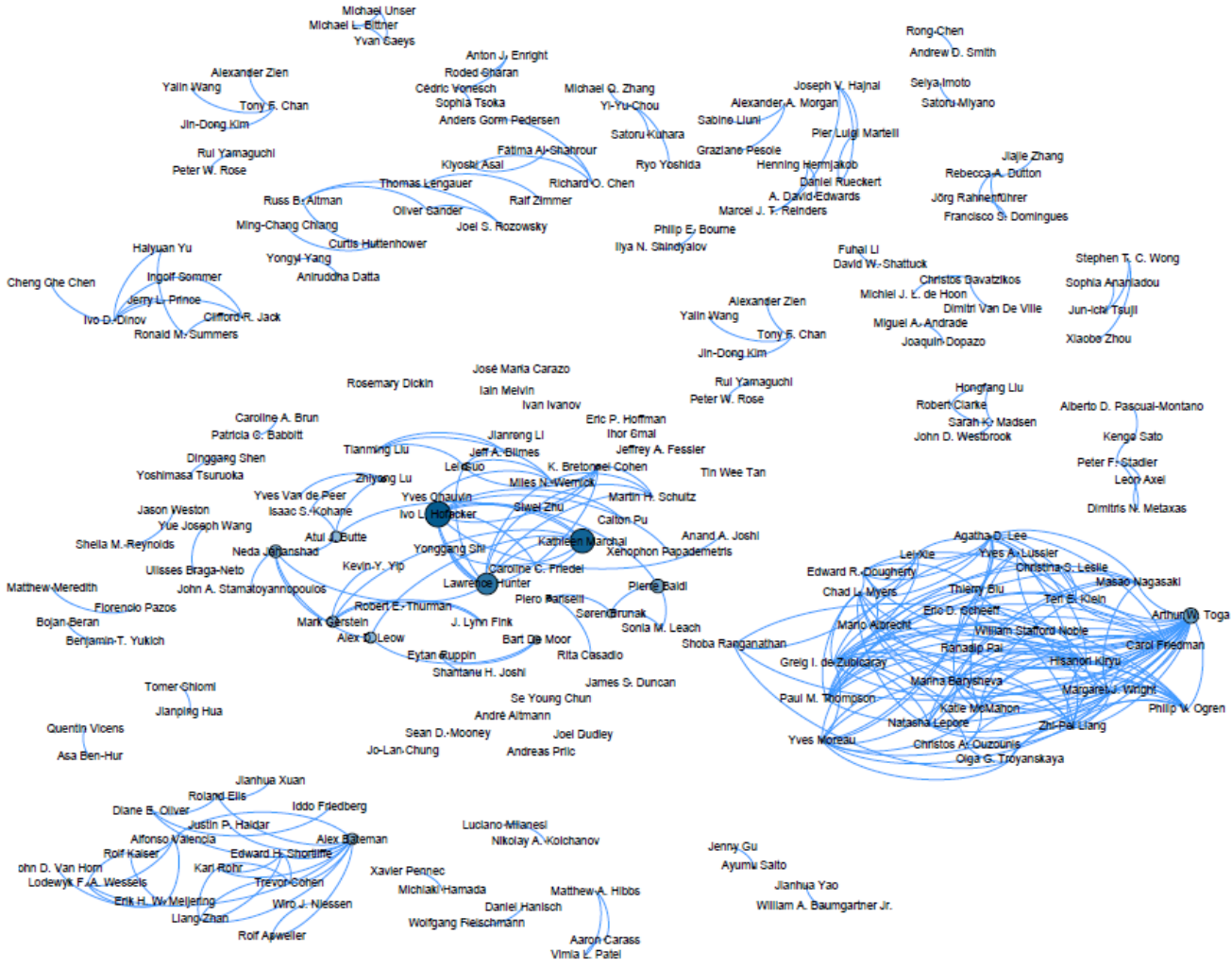

Supplement: Supplementary file 8 — Authors’ original file for figure 8 [file 40064_2012_267_MOESM8_ESM.pdf]
